# Disruptive Behavior in the Postdisciplinary Society

**DOI:** 10.3389/fpsyg.2021.740856

**Published:** 2021-09-14

**Authors:** Joaquin Gaete-Silva, Alfredo Gaete

**Affiliations:** ^1^Werklund School of Education, University of Calgary, Calgary, AB, Canada; ^2^Calgary Family Therapy Centre, Calgary, AB, Canada; ^3^School of Psychology, Universidad Adolfo Ibáñez, Santiago, Chile; ^4^Campus Villarrica, Pontificia Universidad Católica de Chile, Villarrica, Chile

**Keywords:** disruptive behavior, normalization, inclusion, justice, well-being, diversity, conflict – interpersonal, conduct (behavioral) problems

## Abstract

Responding to disruptive behavior has become increasingly problematic in current Westernized societies, impacting people’s well-being globally. In the context of the current Special Issue, in this article, we advance the concept of *problematic disruptive behavior* (PDB) as a suitable “window” to better understand some aspects of the deep interdependence of social participation, citizenship, justice, and well-being. To do so, we also advance the notion of *postdisciplinary society* to account both for the apparent rise of problematic disruptive experiences, and the increased social conflict within which such experiences get often entangled. More specifically, we argue that formerly morally acceptable responses to problematic disruption, such as punishment and discipline, have lost social legitimacy and, to that extent, they aggravate the problems they were intended to resolve. We provide a genealogical account of the surge of such postdisciplinary order with a focus on the moral transition on ideas of justice, of personal entitlements, and authority. We conclude outlining an alternative way to respond to disruptive behaviors that we anticipate will be both more effective and acceptable in the current postdisciplinary milieu.

## Introduction

*Disruptive behaviors* (DBs) can be roughly defined as actions breaking social norms (Gaete and Gaete, 2021). A recent Google search on this label using an incognito window yielded over 450,000 results, the top 10 consistently including a vocabulary with negative connotations. It is used to refer to cursing, interrupting, harassing, bullying, threatening, hitting, stealing, lying, and other “socially inappropriate,” “offensive,” or “harmful” actions. Headings and subheadings within these results treated people’s disruptive doings as problems to be faced or solved, by means of expressions like “how to deal with…,” “how to manage…,” etc. For clinical psychologists and educators alike, the experience of negative disruption seems to be mushrooming, particularly with respect to young populations (e.g., Nock et al., 2006; Rivenbark et al., 2018; Education Advisory Board, 2019). In the United States, teachers report an “alarming increase” in disruptive behavior, estimating that it is exhibited by nearly one-quarter of their students (Education Advisory Board, 2019, p. 4), highly impacting teachers’ job satisfaction (Sims, 2020; Toropova et al., 2021).

To say that DB affects disrupted parties’ (e.g., teachers’, parents’) well-being worldwide may be to state the obvious. Perhaps less self-evident is the toll of disruption on disruptive parties, supported by researchers using a variety of methods and theoretical orientations that deem DB as both cause and consequence of enduring personal distress (Freud, 1957; Gilligan, 2001; Colman et al., 2009; Erskine et al., 2014; Issmer and Wagner, 2015; Frick, 2016). Notably, in their 40-year follow-up, massive longitudinal study, Colman et al. (2009) concluded that adolescents in the United Kingdom that were experienced by their teachers as disruptive when they were 13–15years old, by the time they were 36–53years old had an increased risk of alcohol abuse, clinically meaningful distress, along with various forms of relational, educational, and financial difficulties (Colman et al., 2009).

Now, not all disruptive actions are regarded as negative or problematic, and some may even be seen as virtuous and desirable. Think in some of the (highly disruptive) doings of Marie Curie, Martin Luther King, Fritz Perls, Vicente Huidobro, Frida Kahlo, and a practically endless list of both celebrities like these or relatively unknown people whose disruptive deeds have nevertheless been widely welcomed and celebrated by a significant portion of humankind. Elsewhere, we have already noted that DB cannot be reduced to *problematic* disruptive behavior (PDB) and accounted for the latter as a matter of *affiliation discapability* stemming from conditions of social injustice (Gaete and Gaete, 2021). A more traditional approach would explain it as the expression or effect of “mental disorders,” in the sense in which this expression is commonly used in psychiatry (see, e.g., American Psychiatric Association, 2013).^1^

But regardless of either of these two accounts, neither of them tells the full story. For some, disruptive actions are problematic simply because they are experienced as such, and also because those experiences may yield quite problematic interpersonal difficulties, as Garfinkel’s (1984) so aptly demonstrated in his now classic breaching experiments.^2^ PDB at school provides an illustrative example. Imagine a teacher experiencing a student repeatedly refusing to be on task. Perhaps the student keeps looking through the window and ignoring what the teacher says, perhaps even mocks the teacher, showing no respect. Many teachers will experience these actions as disturbing wrong doings. Likewise, the student may resent his teacher’s demands as morally unwarranted. Perhaps the student thinks it is right to resist a corrupt institutional order (see, e.g., Jackson, 1990). At any rate, both parties will treat each other’s actions as wrong due to breaching a tacit norm. Not unfrequently, this two-way street disturbance, where each party feels entitled to do what they do and finds the other doing something inappropriate, will give rise to struggles that may escalate and become deeply detrimental to them and related stakeholders (e.g., the student’s parents, classmates, classmates’ parents, school principal, and so forth).

Thus, the P in PDB can refer to at least three different issues: (a) a certain explanatory narrative, (b) a disturbing experience associated with a moral evaluation, and (c) an interpersonal conflict (originated around a certain disruption). Or, if preferred, we can say that there are at least three (compatible and possibly related) ways to classify a disruption as problematic: (a) in virtue of its etiology, (b) in virtue of its phenomenology, and (c) in virtue of its social consequences. This article focuses on PDB mainly in the last two senses.

People seem to respond to disruption by engaging in rather problematic interaction patterns (Patterson, 1982; Baumrind, 1991; Omer, 2011; Besnard et al., 2013; Tomm et al., 2014; Yan et al., 2021). PDB has become increasingly problematic during the last decades in this sense, and the central purpose of this article is to account for this recent growth of (c) PDB’s conflict-problematicity. But, we believe that the increment is related to a moral aspect involved in (b) the phenomenological problematicity of PDB, which in turn is related to a major social and moral shift that occurred in the last few decades and gave birth to what we have called here *the postdisciplinary society*.

Our main argument is that PDB has become more problematic because current Westernized (e.g., pluralist, multicultural, secular, liberal, and democratic) societies across the globe are undergoing a deep moral transition associated with an unfolding idea of social justice. We describe it as a transition from a disciplinary to a postdisciplinary society, and try to show how it has created conditions for PDB proliferation (in the second and third sense above), thereby significantly compromising people’s welfare across the globe. More specifically, we argue that formerly morally acceptable responses to (b) experienced disruption, such as punishment and discipline, have lost social legitimacy and, to that extent, they (c) generate further social conflict, aggravating thus the problems they were intended to resolve. First, we describe the main aspects of the disciplinary and the postdisciplinary society, to show how the passage from the former to the latter involved a major change in the way to demarcate the socially just, and how this change rendered traditional approaches to deal with PBT both unacceptable and inefficient. We also suggest a different approach that in our view offers a better suited way to respond to PDB in the postdisciplinary society.

## The Disciplinary Society

According to Foucault (1995), at the beginning of the 17th century Europe went through a major shift in how social control was to be accomplished. Many states deemphasized the use of brute force and military power to gain obedience, as it became increasingly inefficacious: too expensive to manage large masses of people. In addition, emerging humanist and egalitarian ideas made punishment involving unnecessary pain (e.g., torture) highly controversial from a moral point of view (Taylor, 1989, 2009).

Prompted by these changes, a whole new variety of methods for social control came to dominate the European scene. Foucault (1995, p. 170) famously called this *disciplinary power*, or the “art of correct training” to bring about docility and, ultimately, productivity and capital. The growth of disciplinary training was so spectacularly key to modern social institutions (e.g., the new industry and its new market, the modern prison, the hospital, the school, and the modern army), that by the end of the 18th century it had given birth to a whole new form of social organization – the *disciplinary society*.

One of the most important strategies that disciplinary power rests on is *normalization* (Foucault, 1995).^3^ Famous analysis of the modern prison of Foucault (1995) illustrates well how control could be achieved by creating the illusion of being observed at all times (hence Bentham’s term for it, the *panopticon*). Persistent observation allowed the creation of population standards in ways that statistical norms became moral norms: what people typically were able to do under sustained supervision begun to be treated as what the same people were allowed to do without supervision, and then what people now branded as “normal” were minimally expected to do (cf. Hacking, 1990).

The next step in the development of disciplinary power is to render normalization a *technology of the self*. Critical analysis of Foucault (1988, 1995) suggests that norms become appealing to individuals themselves. Rather than feeling forced or coerced, individuals experience themselves as “freely” choosing to behave in accordance to the normal. Once the norm is enfolded into the person (i.e., internalized – see Rose, 1996; see also Parsons, 1968)^4^ it becomes a form of self-control – a continuous form of mild self-reprimand that fits with the modern self-understanding of being a free agent. The Kantian idea of freedom as self-regulation is used and abused as the ideal served by this disciplinary device. As Freire (2005) would put it, people introject their dominator – usually, and mainly, through schooling (i.e., by means of disciplinary practices).

A modern idea that complements description of the disciplinary society of Foucault (1995) is the idea of people as free agents equals in dignity. This is a modern notion in the sense that it became essential to the way in which people conceived of themselves and their world in modern societies, especially from the rise of (modern) democracy and the first nation-states onwards. In contrast to pre-modern self-understanding, which was based on a transcendently given cosmic order, society came to be imagined rather instrumentally, as a kind of freely consented contract between equals (Taylor, 2004; see also Rousseau, 1998). The promise of a society aimed at a mutual benefit, *via* normalization, becomes thus the new hidden but highly efficacious order coordinating great masses of disciplined, self-regulated individuals oriented to develop to the best of their capacities.

We endorse thesis of Foucault (1995) on normalization as a strategy to manage PDB within the disciplinary society. Colonizing practices present themselves as a paradigmatic case, in which normalization’s disciplinary training is seen as the right thing to do regarding “other” cultures – to the extent that failing to colonize the other was perceived (by the potential colonizers) as a form of undue harm or neglect. Norms *need* to be socialized, ideally by humanitarian means ultimately seeking rational consent. To the colonists, disciplinarian methods such as instructing, preaching, modeling, confronting, or any other means to appreciate the superiority of their norms are fair: it is morally acceptable, even admirable, to “make” the colonized understand, accept, learn, have an insight, correct course, and ultimately join the cause. And perhaps not so ideally, socializing agents will legitimately impose rational norms to those unable to understand but who need to comply.

To inscribe the same norms in every person’s soul was seen as a way to produce a *just* society – one in which each individual is equal in a rather brutal way, insofar as each had been made of the exact same normativity. Equality among citizens, expressed in the ideals of the French Revolution, and mediated by normalization, became the heart of the modern conception of social justice for the next 2 centuries. Nonetheless, rather than securing genuine democratic equality, the normalizing/disciplinary society ended up paving the way to the homogenization of people (Gaete and Luna, 2019) in a world inhabited by “similar, but unequal individuals” (Touraine, 2000, p. 10) – a world very much like Orwell’s animal farm, where some animals were “more equal” than others. In the end, assimilationism and colonialism took over egalitarianism.

## Postdisciplinary Resistance to Normalizing Practices

“The operations that one would have to perform in order to produce and sustain anomic features of perceived environments and disorganized interaction should tell us something about how social structures are ordinarily and routinely being maintained” (Garfinkel, 1984, p. 187).

The last turn of the century brought a strong social and political reaction to modern democracies’ failure to materialize their core ideals (Gaete and Luna, 2019). People started to resist disciplinary society and its monolithic view of a single normative order, which nowadays is felt as both a limiting and illegitimate imposition. By *postdisciplinary society*, we mean here this form of resistance, animated by ideas and sentiments that have spread almost globally. One of its central motives is that of a diverse world, where different but equally legitimate forms of life coexist respectfully – an ideal that can be wholly or partially detected in several recent social changes. Take, for instance, the explosion of communities facilitated by the development of communication technologies, which have contributed to the multiplication of meaning-making contexts and intelligible identities (Gergen, 1991, 2009). Consider also the proliferation of cultural, religious, gender, and sex orientation claims for acknowledgement (the image of the rainbow is powerful: a colorful world with different communities claiming their equal share for participation in social life); or the inclusive movement in education, which includes the celebration of a *diversity of diversities* in a framework of equal rights and opportunities (Booth and Ainscow, 2002; Gaete and Luna, 2019).^5^

This “diversity turn” of the postdisciplinary society came hand in hand with a significant shift in the conception of justice. The disciplinarian notion of justice as a matter of mere equality was too narrow to accommodate the demands on the recognition of difference (e.g., Honneth, 1996; Taylor, 1997; Fraser, 2003). Early proponents of identity politics as highly relevant to psychology articulated a similar view (e.g., Sampson, 1993). In the face of multiple claims for recognition, justice had to be expanded beyond mere prosperity and mutual benefit (e.g., equity and redistribution) to include the right to be who one is. In this vein, Taylor (2009) suggested that an ethic of authenticity “has utterly penetrated popular culture only in recent decades” (loc. 6,764), thereby resisting the disciplinary ethic of order and progress. No matter how different a form of life happens to be from those whose *habitus* (Bourdieu, 1977) used to set “the norm” in disciplinary societies, they are entitled to be acknowledged as full citizens. In this view, norms and other forms of “institutionalized patterns of cultural value” are considered to be illegitimate if they render some individuals “inferior, excluded or simply invisible” (Fraser, 2003, p. 89). There is no justice without respecting identity, and no respect for identity without *participatory parity* (Fraser, 2003; see also Fraser and Butler, 2016).

In culturally embracing participatory parity as a demarcation of the socially just, people and institutions that once were uncontroversially disrupted parties are rendered disruptive. The school is an illustrative example. For a long time, this institution encompassed a series of practices widely legitimated by social actors. Those who dared to defy them, as many students tended to do, were (and still are) casted as disruptors and, rather unproblematically, often diagnosed with a “mental disorder” (e.g., a “conduct disorder,” an “oppositionist disorder,” or, more subtly, an “attentional deficit disorder”). But with the coming of postdisciplinary resistance, the school and its usual practices have started to lose legitimacy, and students previously considered disruptive have started to be seen as the disrupted parties of such illegitimate institutional practices.^6^ Now classic work of Jackson (1990, p. 1) on life in school classrooms points to this from the very beginning with a quote from Roeke: “The ‘order,’ the trivia of the institution is, in human terms, a disorder, and as such, must be resisted. It’s really a sign of psychic health that the young are already aware of this.” The problem, the disorder, is not located within the student any more. It is the institutional “order” that stops being considered as such, as many others made it clear during the second half of the last century (see, e.g., Illich, 1984; Pink Floyd’s *The Wall* is a piece of pop culture signaling the same phenomenon).

Arguably, those who stick to a disciplinary conception of society and justice (or the internalized disciplinarian in some of us that still finds comfortable with it from time to time) may experience postdisciplinary resistance as highly disruptive – perhaps too nihilist (“anarchists!”), perhaps too sensitive (“I’m walking on eggshells!”). Educators and agents engaged in traditional disciplinarian roles indeed feel at a loss regarding how to manage PDB (Omer, 2011; Education Advisory Board, 2019), particularly in culturally diverse settings (e.g., Glock et al., 2019). Nonetheless, what we suggest here is that postdisciplinary claims for participatory parity are ceasing to be a resistance and starting to be the moral foundation for a new social order structured in a new form of democracy (see also Gaete and Luna, 2019). For us, the ubiquitous breaches, how masses of people seem to be treating formerly unproblematic normalizing practices as if these were not only groundless but offensive, is the clearest proof of a new moral order at issue. To use language of Garfinkel (1984) in the quote above, the reactions to disciplinary power might be held as the type of “operations” telling us something about new social structures at issue.

In all, identity politics led by a few enlightened activists during the second half of the 20th century (e.g., civil rights and gender movements across the globe) pioneered the now widespread, hyper inclusive, participatory postdisciplinary spirit. Asserting the legitimacy of different forms of life went from an almost unintelligible idea to a movement, to a taken-for-granted way of experiencing the world – a new natural attitude (Schutz, 2010). For the first time in human history, a significant portion of the world population feels attracted to a moral outlook according to which all people, no matter who or how many they are, can and even must fully participate as citizens in the construction and destiny of their polis. In our view, this emergent postdisciplinary society has created conditions for constituting new agents of potential disruption, and hence for a significant rise in PDB. And the quantitative surge of disruptors, in addition to cultural anomy, creates conditions for sustained interpersonal conflict. Let us elaborate on all this next.

### New Disruptors

“One angry rebel is crazy, three is a conspiracy, fifty is a movement” (Tavris, 1989, p. 262).

Problematic disruptive behavior can be seen as a form of intersubjective activity or co-action (Gergen, 2009), in the sense that it takes at least two people for it to happen and a partially shared world of meaning. More specifically, for someone to be effectively (i.e., hearably, noticeably) disruptive, the breaking of the norm needs to be acknowledged by another party as such, which requires that this other party sees the norm breaker not only as someone who should observe an assumedly shared norm, but also as someone who *can* break norms. But this is a prerogative of citizens. Nowadays one can easily neglect this, especially if one lives in a postdisciplinary society and, consequently, is rather used to the postdisciplinary idea of social justice, according to which everybody gets to hold citizenship to some extent. So everybody is a potential norm breaker. But things were not always like this. For the sake of contrast, think of life in ancient Athens, where only a handful of people were acknowledged as citizens. Barbarians, for example, had no chance to hold citizenship and, to that extent, were not able to disrupt Athenians’ life in this sense. The sole actors that could effectively disrupt the social order were recognized citizens. Barbarians were able to disturb Athenians’ life in several ways (e.g., by invading the city), but they could not be disruptive to them in the way in which fellow citizens could. Athenians simply did not care what Barbarians did (in this sense). They lived outside the polis and they were to stay out of *the polis way* – quite literally, for it was the highway out of town. They were not socially legitimate agents.

Ancient Athens contrasts sharply with modern and postmodern societies. Thankfully, our Westernized societies take seriously the voices, doings, needs, beliefs, hopes, and demands of a broader range of humans, perhaps even of other sentient beings (some are ready to give a certain degree of citizenship to pets, for example). But this goes hand in hand with a substantial increase of disruptive practices. True, historically marginalized groups’ voices are *de facto* still relatively dismissed, but almost no one living in contemporary democracies would dare to say, in almost any context, that such groups *should* be dismissed or ignored. Even non-citizens such as children and newcomers have (again, thankfully) become agents of potential disruption. Their voices are and get to be heard. Greta Thunberg, the 15-year-old who stopped going to school to protest for the climate change (and became Time’s 2019 person of the year), is a case in point.^7^ Perhaps less spectacular than Greta’s planetary disruption is the situation of so many educators and parents who feel that children’s protests seem more hearable than those when they were children (e.g., Hanes, 2014). Even toddlers nowadays are seen as agents of worth attending disruption and, no wonder, defiance to authority seems nowadays to be ubiquitous. So while DB has existed forever, the postdisciplinary extension of at least some degree of citizenship to virtually everybody has increased the number of potentially disruptive people. The multiplication of hearable agents magnifies the chances that behavior formerly dismissed as emanating from “non-citizens” may now be taken (more seriously) as expressing “movements” (as Tavris’ puts it in the quote above) – legitimate to some, disruptive to others. At any rate, it is clear that under these circumstances DB skyrockets.

Plus, technology has made recognition of such movements readily available, and when historically marginalized groups manage to gain recognition, norms are multiplied and interlocked kaleidoscopically. Communities do not only have the right to be (“identities”); their existence has become feasible as armies of supporters (“followers”), which are now at a click of distance. Now, as Taylor (1989, p. 3) claimed, identities and morality are “inextricably intertwined themes.” Communities of reference legitimized by its own members proliferate, and with them, each of their constitutive moralities are advanced – their codes of acceptability, their “shoulds.” Agents hearably claiming recognition, participation parity, and respect as a matter of identity, and proliferate. In all, as communities and their felt entitlements are thus multiplied, so is the potential for someone to feel offended by others breaching those codes and/or illegitimately imposing their own. Conditions are set for a surge of disturbing experiences associated with moral evaluations – which is what in the introduction we distinguished as PDB in the second sense.

### Cultural Anomy

In a postdisciplinary society where groups demanding equal recognition abound, trying to make everyone the same (“homogenizing difference,” to take an expression from Taylor, 1997, p. 61) will not do. Thus, in order to resist the attempt of assimilating all forms of life to the practices prescribed by a unique, taken-for-granted code regarded as “superior” (as it happened in the disciplinary society), some will be tempted to juxtapose all of the existing codes – to place all cannons and standards at the same level of correction, so to speak. When no principle can be chosen as better suited than others, one can easily fall into the feeling that one has no clue to distinguish the true from the false, the right from the wrong or, perhaps less dichotomically, a preferred pathway forward. We call cultural anomy this disorientating sentiment ensuing from the mere juxtaposition of epistemic and moral codes.^8^

An example of cultural anomy in the moral realm is the enormous confusion some parents undergo when their children diagnosed with “attention deficit hyperactivity disorder” engage in PDB. They are told (by a mental health professional, the school principal, a worried relative, etc.) that they can mitigate the kid’s disruptions by “medicating”^9^ them. While many parents may be at peace with using drugs to manage problematic disruption (or “treat” their children’s “mental disorders”), many others may feel much more conflicted about such practice and may experience cultural anomy. Perhaps they feel at a loss, as they struggle finding acceptable grounds to establish a hierarchy between observing, say, a principle like “do not hinder due medical treatment to your child,” and some other conflicting principle like “do not take drugs to enhance your performance” or “Never use hard drugs with children.” Whether it is because they cannot establish a hierarchy or because they have been simply seduced by code juxtaposition and moral relativism, the point is that they are unable to see the right thing to do (perhaps not even whether there is a right thing to do).

But these disoriented parents may have to deal with yet another difficult decision, namely, that of whether their children’s disruptive actions are in fact illegitimate. Even if the actions break certain norms they have been assuming to hold, their children may not share that same code. Again, in a world in which everybody is entitled to choose their own code, parents may feel that they have no right to impose a code on their children. After all, they might be wrong – and they know it. They may be quite certain, for example, that at least some of their children’s disruptions are legitimate reactions to some questionable, psychologically violent actions of their own (or of their children’s teachers, etc.). They may also know that the drugs they think can help them might also have secondary effects, which could end up damaging their children; or, alternatively, that by not “medicating” them they are potentially exposing them to school failure and even to some dangerous situations. How, then, can they make a decision here, when they are not certain about the one thing they need to be – their decision?

A similar disorientation can occur in other contexts; for instance, when teachers must face their students’ PDB, or when couples must face some of their deeper (e.g., moral) differences, or when societies must face demands for recognition. For the purposes of this essay, the point we want to make is that while the proliferation of disruptors and their codes may account more directly for the quantitative rise in PDB (i.e., “P” in the second sense), postdisciplinary cultural anomy may explain why disruption has become qualitatively harder to handle and why more than ever it may be triggering and sustaining social/moral conflict (i.e., the “P” in the third sense). For it makes the task of discriminating the legitimate from the illegitimate not only more frequent, but also harder to accomplish. We would think that for many digital native Millennials (Prensky, 2001) one-size-fits-all moral frameworks are eo ipso experienced as arbitrary, impositional, and a form of unacceptable colonization if not of personal offense. One group’s identity/code may be experienced as offensive to another’s. Donald Trump’s presidency in the United States has epitomized how nationalisms seem to be increasingly disruptive, but not unpopular, in a postdisciplinary society. The disruption recently demonstrated by Chilean constitutionalists protesting during a live performance of the Chilean national anthem at the opening ceremony of the constitutional convention can be seen as another example of the same trend – many condemned it, many praised it, many have no idea what to make of it.

Part of the difficulty here may be technical: instead of effectively handling PDB, traditional responders may feel that they only escalate in conflict. Former effective disciplinary methods such as punitive and exclusionary strategies beget only more turmoil and conflict (Patterson, 1982; Baumrind, 1991; Borum et al., 2010; Omer, 2011; Osher et al., 2014; Gaete et al., 2020; Gaete and Gaete, 2021). Whether it is imposing alien preferences coupled with disregarding such views, criticizing coupled with defending, or blaming, accusing, and attacking coupled with counterattacking, such interpersonal patterns of mutually triggering behaviors may arguably acquire life on its own resulting in increasing appearances of PDB (see Tomm et al., 2014; Sametband and Strong, 2018). No wonder teachers often report an increased sense of unreadiness to manage discipline in the classroom, impacting their job satisfaction (e.g., Gaete et al., 2016; Toropova et al., 2021).

Nonetheless, we hold that this sense of cultural failure is not primarily technical but ethical. Disciplinarian methods have turned ineffective because they have become morally unacceptable. In Modern democracies, punishment in almost all its applications, and a good deal of disciplining methods (especially when lacking express consent), now trigger remorse on one side and resistance on the other. Omer (2011), for instance, suggested that technical inefficacy in managing what we here call PDB relates to anomy linked to the fall of a role-bound authority model. Old school appeals to role (“Do it because I am the teacher and you are forced to do as I say”) have become not only ineffective, but morally illegitimate to handle PDB, and a recipe for escalation in conflict – a battle the teacher is doomed to lose, precisely because the disciplinary tools they once could resort to have become increasingly unethical and thus are no longer available. Teachers experiencing everyday classroom disruption are a paradigmatic case in point, but we hold that a similar anomic (and chronically stressful; see McEwen, 2017) predicament applies to parents, caregivers, health workers, and to some extent to every fellow citizen who feel at a loss vis-à-vis how to deal with PDB not just effectively, but acceptably.

While the technical and ethical aspects of cultural anomy may be conceptually separable, we see these as rather inseparable in actual attempts to manage PDB. In our view, punishment first, and more recently disciplinary methods, are becoming objectionable as profound changes in our (moral) ideas of justice continue to unfold within the postdisciplinary society. Shortly put, formerly acceptable disciplinary methods themselves have become disruptive. In order to fully appreciate this subversion, zooming into the phenomenology of PDB seems warranted.

### Responding to Disruption in a Postdisciplinary Society

Problematic disruptive behavior is an intersubjective phenomenon embedding an evaluation: disruption is experienced as “wrong.” Now, responses to PDB can be said to express not one, but two tacit evaluations. For, in addition to the assessment embedded in the experience of disruption, there is an assessment entrenched in the person’s stance toward the experience of disruption; a meta-assessment evaluating the legitimacy of the assessment itself (e.g., “it is right to experience this as wrong”).^10^ Accordingly, we would like to distinguish three stances expressed in the way that a person responds to experienced disruption. A person may respond assuming their tacit first assessment (“it feels wrong”) is immediately legitimate. We call this stance *immediately hierarchical*,^11^ as the respondent to disruption takes for granted that their first (tacit) assessment is more legitimate than or “superior” to any competing interpretation of the situation at issue. Alternatively, a person may adopt a stance of *immediate juxtaposition*, treating both their own and the disruptor’s initial assessments as equally legitimate, accepting the other’s tacit preference at face value. Finally, a person may choose to take a *tentative* stance, by deferring the stronger, meta- or second evaluation. The person may deliberately assume that, at the time of their felt disruption, judging the other’s behavior as wrong might be too partial, inadequate, or even failing to regard the other person as a full partner in interaction.^12^

Now, both the immediately hierarchical and the immediately juxtaposing stance seem to be unfit for a postdisciplinary society. In adopting the former, disrupted parties automatically experience the disruptor’s behavior as wrongful and their own evaluation as right or appropriate. The feeling of wrongfulness, or *de facto* preference (first evaluation) is taken thus at face value as a normative preference (meta- or second evaluation). The other person’s behavior is experienced as an illegitimate protest and treated as such upon reflection, assuming that one’s own perspective should be regarded as the more (if not the sole) legitimate one. Returning to our paradigmatic case, the colonists simply take for granted the superiority of their own worldview. Perhaps invisible to them as *a* worldview, colonists may take their ideas and principles as a mirror of just how things objectively are or should be. Their allegedly self-evidently “rational” norms/standards are not to be problematized but educated in and conformed to. In contrast, in an anomic postdisciplinary society, fueled by an expanded notion of justice as participatory parity, normalizing efforts revealing a stance of immediate hierarchy are increasingly experienced as involving illegitimate impositions – which, as we saw, brings about further PDB. Former educators are pervasively interpellated as if they were disruptive pupils.

This is not to say that all people taking an immediately hierarchical stance will necessarily engage in normalizing practices. People may think they are right in judging others’ behavior as wrong, but pragmatically decide not to engage in what they realize are rather ineffective or even counterproductive methods to manage problematic disruption. As Watzlawick et al. (1974) famously put it, people may realize that sometimes the solution is the problem. People may strategically decide to avoid escalating by containing rather than solving an interpersonal conflict. They may yield to the other’s views partly or fully, while still believing the other’s behavior to be normatively wrong (and thus adopting a stand of immediate superiority). Several variants of such hierarchical stance may be taken in response, from deliberately deciding not to persist arguing (e.g., leaving the field of interaction), blaming third parties (e.g., triangulating, scapegoating; e.g., Bowen, 1993; Girard, 2014), deciding to “agree to disagree” (the quotation marks here signal the agreement does not reflect true understanding of the other’s views, or that understanding is little more than lip service). Regardless, we see all these hierarchical variants as postponing or hiding rather than aptly facing moral conflict, and thus as relatively unfit to manage diversity in a postdisciplinary society.

Now, defaulting to a juxtaposing stance seems to be problematic both in a disciplinary and in a postdisciplinary society alike. In the former case, responding to PDB by instantly assuming that two competing views are equally valid is simply irrational; and it is unfair and wrong to hold that all views, no matter what they are, are equally legitimate. An “everything goes” stance is at odds with one of maximizing social prosperity or “mutual benefit.” On the other hand, mindless validation in a postdisciplinary society, so preoccupied with mutual recognition and respect, will not do either. For, arguably, one cannot find true value in something one does not even care to understand. Following suggestion of Taylor (1997), we predict that such a stance will be experienced as shallow, patronizing, and disrespectful, generating further disruption. Genuine recognition of the value of others’ views occurs after, not before, such views are known and understood.

Given that immediate hierarchy and juxtaposition may seriously compromise well-being, justice, and citizenship in a postdisciplinary society, what alternative may a psychology for the common good embrace? How is psychology to face the great challenge of promoting justice as participatory parity, expressed as virtuously responding to PDB that may express diverse moral views? Although, answering these questions in detail clearly goes beyond the scope of this article, we sketch two features of a more promising stance that hopefully will be expanded in future inquiries.

### Bracketing With Transforming Tentativeness

Instead of *immediately* assuming either hierarchy or juxtaposition of competing views/codes, we suggest that a more suitable way to approach PDB (or sustained interpersonal conflict) in a postdisciplinary society is to adopt a stance of deciding not to decide too prematurely who is right. By “bracketing” or taking tentatively the judgements embedded in our *de facto* feelings (e.g., “it feels wrong”), this stance deliberately defers committing to a normative assessment of our and the other’s disruptive experiences. We would think such a tentative stance affords both validating immediate experience-as-experience (i.e., first evaluation of PDB as “it feels wrong”), while bracketing the stronger, full-fledged moral evaluation (“I *should* feel this as wrong”). Informed by a postdisciplinary, shared-yet-plural social world, such tentativeness may allow parties in conflict to acknowledge actual disruptive experiences as possible and intelligible, while opening space to work out a fuller, more inclusive, mutually acceptable account of a common good. Far from indifferent or “neutral,” then, we see such tentativeness shaped by a principle of charity, i.e., by a temporary assumption that the other person may have good reasons to behave the way they do, at least in some respects – very much like a translator may charitably assume foreigners are probably telling the truth when attempting to understand what they say (Aristegui, 1999).^13^

We will call this a stance of *transforming tentativeness*, as we envision it not only as temporarily assuming relative ignorance of moral content, but also as involving an openness to eventually transform one’s moral framework in the light of the other’s. This, of course, means being ready to transform oneself, at least to the extent that our moral framework is a fundamental aspect of who we are. We see such readiness as informed by, the idea of justice as participatory parity (Fraser, 2003), and thus as a better fit for a postdisciplinary moral outlook built upon justice so construed.

Note that the transforming aspect of tentativeness is not at odds with establishing and endorsing hierarchical or juxtaposing relations between different moral views. Actually, once two or more people decide to deal with their differences by taking this stance, it would be possible for them to end up embracing a hierarchical or juxtaposing stance anyway, among other possible outcomes (we elaborate on this shortly). But unlike going *immediately* hierarchical or juxtaposing, in transforming tentativeness people can reach moral hierarchy and juxtaposition as joint accomplishments; more specifically, as the result of a participatory form of inquiry (rather than as a matter of unreflected starting points in their experiences of disruption; cf. Garfinkel, 1984). We are not suggesting, then, to disregard our immediate experience but, on the contrary, to take our spontaneous or “natural” attitudes to disruptive experiences with curiosity, less as mechanically caused by others’ behavior and more as what makes sense within a certain moral framework or form of life. Further, our experiences of disruption can be deemed as suitable for modification within a more inclusive moral “game,” where others’ behavior is not necessarily interpreted as breaking the rules of “our” game (cf., Wittgenstein, 1953; Garfinkel, 1984). Disruption may thus be explored charitably, as a participatory endeavor aimed at developing greater confluence (Gergen, 2009), and as an opportunity to learn something that may be important to us-parties-in-conflict. Unpacking in detail how such participatory forms of inquiry could be conducted exceeds the scope of this article, but in the discussion section, we acknowledge some notable contributions that could be taken as a promising starting paradigm. In the remainder of this section, we would like to sketch three scenarios that may result from accomplishing either hierarchy or juxtaposition, which we see as more adequate forms of responding to PDB in a postdisciplinary society.

The easiest scenario would probably be one in which parties jointly accomplish hierarchy. This may happen after listening to each other’s point of view within a less constrictive or adverse relational environment, facilitated by transforming tentativeness as described above. For instance, deliberately deferring judgment may help co-creating a way of relating between parties in conflict that *eo ipso* facilitates mutual listening and understanding. Instead of hearing the other’s utterances as a form of criticism (inviting, say, defensiveness and closure to understanding the other’s experience and views, rather than effective or compassionate listening), the other’s inquiries may be heard as validating experience-as-it-was-experienced, optimizing thereby the articulation of the speaker’s sharing of their experience (see e.g., Tomm et al., 2014). Perhaps a party comes to admit an offense (e.g., “lying”), when both parties come to treat one another with respect – or when they agree that the offense gives no reason for the offender to be humiliated. In such a scenario, tentativeness can be said to be transforming in a procedural sense: by deferring strong evaluation, a negative relational environment (e.g., humiliation inviting defensiveness and vice versa) can be prevented, facilitating thereby the accomplishment of agreements vis-a-vis behavioral standards (i.e., accomplished hierarchy).

In such a scenario of *accomplished agreements*, we anticipate that parties will welcome rather than resist normalizing methods. When parties have enough competencies to conform to mutually accepted norms (e.g., “being rude is wrong”), kindly reminding (“confronting”) future breaches will suffice to stop escalation. When parties seem to lack the required and mutually desired skills (e.g., “communicating one’s needs assertively”; “standing for one’s rights”), some form of teaching or plan for competency development will likely be welcome as well. Norms will thus not be experienced as alien impositions, and the likelihood of future experiences of disruption will decrease significantly. Parties of course often come to agreement on certain aspects of their conflict (e.g., agreeing on substance but not in form), in which case parties will probably welcome disciplinarian/normalizing methods in those areas of agreement.

In relation to scenarios, where parties accomplish juxtaposition – situations where parties gain some clarity on areas of continued disagreement – a more suitable relational environment may help parties in conflict reach a deeper understanding of one another’s perspectives. The disrupted person may come to regard the disruptive person’s views as legitimate, but different than theirs. A paradigmatic example may be the situation of a couple parenting children engaging in what the parents experience as highly disruptive, self-harming behavior. Both parents may agree that harming oneself is “wrong,” but they may have quite different ideas of what parents should do in response. Perhaps one partner values virtue and respect for authority over subjective happiness, whereas the other privileges exactly the opposite. They may also agree that their conflict only increases their child’s dangerous disruptive behavior. Facilitated by transforming tentativeness, each partner may come to understand and accept that they weigh their values differently, accomplishing thus juxtaposition: they understand their partners’ preference as legitimate (but not preferred to them) and, in so doing, they may decide to compromise. We would call this scenario one of *compromising tolerance*, and describe it as one in which tentativeness transforms not only proceedings, but the shape of their local moral order. Their accounts or views on what they regard as common goods gets expanded.^14^

Alternatively, parties accomplishing juxtaposition may respond to PDB by what we could call *celebrating difference*. In contrast to compromising tolerance, in celebrating difference there is no pain, but eudaimonic joy. And part of the joy comes from realizing that celebrating and creatively including particular views emerging from different forms of life, different common good horizons further enriches rather than constricts all parties at issue. It is creative, for parties may feel the need to develop new languages and practices to bring forth and realize the new, inclusive, and mutually preferred ways of relating they are contributing to forge – a fuller story, with no distortion of each parties’ original contributing horizons.

In addition to a change in proceedings and abstract ideas vis-a-vis legitimacy, tentativeness in this case becomes fully transforming in the sense that it changes parties very souls (or “selves”; Taylor, 1989). On the one hand, it changes parties moral preferences: parties mutually accomplish a fuller account on what should be preferred in situations like those initially triggering problematic disruption. On the other hand, and possibly with time, it may transform parties’ experiences of disruption themselves. For authentically changing one’s moral preferences typically entails changing what we are prompted to strongly pursue or resist; and this is how we become who we are, including how we spontaneously respond to our meaningful surroundings. In time, the formerly disruptive may become hardly noticeable in some cases, perhaps a source of praise in others.

Philosophers lik Taylor (1997) and Gadamer (2004), among many others, have called attention to this crucial aspect of human interaction, which allows us to create new worlds whenever the already existing worlds have become too narrow. By “fusing” our horizons of meaning, e.g., our moral outlooks, we can give birth to new habitats and in fact new cultures that fit better our daily experiences. We reinterpret, perhaps overcome, the “letter” of our codes, jointly assess our current situation, and co-advance a new, mutually accepted code. Something like this is what happens when families completely reshape their ground rules, when a Congress passes a new Bill, a society comes to write a new Constitution, or when we dropped the modern idea of justice and gave birth to a new, postdisciplinary conception of justice as universal participation parity.

## Discussion

While disruptive behavior may be problematic in virtue of its etiology (Gaete and Gaete, 2021), in this article, we have offered an account on the recent surge of problematic disruption stemming from experiences of wrongfulness, and most of all as generating sustained interpersonal conflict. Drawing on outline of a disciplinary society of Foucault (1995), we have argued that traditional methods to manage PDB have become increasingly problematic in Western liberal democracies across the globe, signaling the emergence of a new moral order – a postdisciplinary society. Expanded ideas of justice-as-participatory parity simplistically assimilated by large masses of people have had a negative impact on well-being, as such ideas have created conditions for PDB proliferation and troubled social interaction.

Acknowledging that delineating a fuller alternative to handle current hyperdiversity exceeds the scope of this article, we have nevertheless outlined an overarching stance of transforming tentativeness. We see our schematic proposal of transforming tentativeness as consistent with the concept of therapeutic alliance (e.g., Muntigl and Horvath, 2014; Flückiger et al., 2018) understood as optimal relational conditions to address both intra- and inter-personal conflict. While, we would think further practices developed within related fields may express similar ethical commitments to not individualize interpersonal conflict (e.g., Winslade and Monk, 2008), our view on transforming tentativeness has been clearly informed by former developments within the field of psychological therapies emphasizing conversation as a means for relational change (e.g., Anderson, 2012; Tomm et al., 2014; Sametband et al., 2017).

We also acknowledge the influence of ethnomethodology/conversation analysis (ETHNO/CA), particularly the notion of epistemic status and stances (e.g., Heritage, 2012) in our view of conversational “tentativeness.” We see ETHNO/CA approaches as both theoretically and methodologically well suited to guide future empirical research on the current Special Topic. In particular, it may help a “psychology for the common good” to develop concrete procedures to guide participatory forms of inquiry aimed at accomplishing hierarchy and/or juxtaposition in the moral domain. Theoretically, both treatment of the so-called problem of intersubjectivity of Garfinkel (1984) and Schutz (2010) seem particularly insightful to understand PDB conceptually. Social actors find themselves in a life-world where they experiment one another as always-already experiencers of the same social world. This fundamental assumption, which is part of actors’ natural attitude (see also Heritage, 1984), is at the very origin of PDB (as we have treated it in this article, as both a phenomenological and a social issue). Methodologically, we see in the ETHNO/CA tradition a suitable approach to make our apt responses to disruption observable and reportable for all practical purposes (e.g., Heritage, 2012). Such fruitful path forward may be one in which empirical research generates useful knowledge in this domain, namely: how participants engaging in participatory inquiries accomplish mutually acceptable emergent moral orders like the three scenarios outlined above, providing detailed descriptions of their (“ethno-”) methods deployed in successfully coordinating moral preferences. And, of course, distinguishing further scenarios may be in order as well.

A complementary pathway might be one of further exploring and further developing existing methodologies already available and apt to navigate the type of highly discrepant accounts on the assumedly shared worlds that seem to be at issue in PDB. In our own professional experience, *reciprocal reflective listening* (RRL; Tomm and Acton, 2011, p. 730) has been extremely useful as a paradigmatic example of such methodologies, and anecdotally, it has served us well as a facilitating tool to deliberately bracket moral judgment and accomplish hierarchy and/or juxtaposition of moral preferences. RRL has been described as a “communication exercise” designed to facilitate mutual understanding between parties struggling with relational conflict, and willing to work out their differences in order to stay in a relationship.

Tomm and Acton (2011) have provided procedural details about how to engage in RRL. In a nutshell, parties in conflict are invited to reflect (i.e., formulate verbally) their listening/understanding in ways that can be acceptable to one’s interlocutor (e.g., “what I hear you saying is….is that correct?”). The most important point of the exercise is to reflect one’s listening as a form of validating the other’s experience – in this case as an (unchosen) experience of disruption. Consistent with our suggested “tentativeness,” the reflecting is performed adopting a humble epistemic stance, for example, by granting higher epistemic rights (e.g., “…is that correct?”) to the conversational partners who are sharing a disruptive experience. Additionally, parties engaged in RRL make a mindful choice for parenthesizing judgment of experience, aiming at “listening to the listening” (p. 734) of the other. They are invited to acknowledge the ontologically subjective experience of disruption, maximizing mutual understanding by deliberately postponing “agreement” (p. 735) with the other person – that is, postponing agreeing with the moral judgment embedded in people’s disruptive experiences. While agreement is not sought for, it is not unusual that it happens as a side-effect of RRL (Tomm and Acton, 2011). This is coherent with the scenario of *accomplished agreement* described in the previous section.

Beyond accomplishing agreements on moral preferences, in our view the ultimate goal of RRL is to accomplish a mutually acceptable understanding of the moral backgrounds at issue – he “games” within which parties’ experiences of disruption make sense. It is only after engaging in several cycles of RRL that parties may accomplish, say, a relative hierarchy and/or juxtaposition of particular norms (goods, principles, norms, values, standards, and rules) that both parties are willing to cast as legitimate, hold dear, and transform their relational lives accordingly. Our schematic proposal is certainly limited and hopefully provisory. We see our participation in this Special Issue as an opportunity to invite others inspired by a “Psychology of the Common Good,” to join us in further exploring and articulating virtuous responses to PDB – as a “window” to better appreciate the interdependence of participation/citizenship, justice, and well-being.

Despite our hopes for concreteness, Aristotle’s long-lasting argument about the impossibility to capture virtue or ethical wisdom (*phronesis*) in a code (*techné*) may be worth mentioning here. First, because the number of potential situations in which a code would need to be adjusted to better serve justice as participatory parity is infinite. Second, because the required virtues are various (e.g., flexibility, courage, compassion, patience, recognition, and solidarity) and, depending on what the situation requires, may conflict one another. The goods at issue in particular situations of conflict may likewise be many (e.g., well-being, equality, autonomy, citizenship, and spirituality). And if Aristotle would have lived in postdisciplinary times, he might have been inclined to accept that accomplishing phronetic assessments gets only more complicated when different groups in society have different views of what the good life is, and what is most important to (multiple) “us.” Nonetheless, Aristotle’s general advice to develop phronesis seems still quite insightful to us: gaining a deep sense of the goods concerned, and the flexible ability to discern the relative weights of such goods in particular PDB situations. General principles or orientations may be of help, but (with Aristotle) we would think that most of all we need to develop some jurisprudence. We think an upcoming challenge for a Psychology of the Common Good might be to promote, notice, collect, and analyze situated examples of actual parties successfully moving from problematic disruption to mutually accomplished local moral orders. In all, our hope is that acknowledging more fully the postdisciplinary society many of us are co-creating may be a step toward co-creating some ethical wisdom in it as well.

## Author Contributions

JG-S and AG have been developing the notion of problematic disruptive behaviors. JG-S wrote the first draft of the manuscript and acknowledges great influence of Foucault’s notion of disciplinary society, Taylor’s moral philosophy, and the discursive psychotherapies. AG commented on and edited the first draft, adding significant contributions mostly informed by his background in Philosophy, Education, and Theoretical Psychology. All authors contributed to manuscript revision, read, and approved the submitted version.

## Conflict of Interest

The authors declare that the research was conducted in the absence of any commercial or financial relationships that could be construed as a potential conflict of interest.

## Publisher’s Note

All claims expressed in this article are solely those of the authors and do not necessarily represent those of their affiliated organizations, or those of the publisher, the editors and the reviewers. Any product that may be evaluated in this article, or claim that may be made by its manufacturer, is not guaranteed or endorsed by the publisher.

## Footnotes

^1^The traditional, psychiatric narrative often depicts PDB as a expressing disruptive, impulse-control, or conduct disorder, construed as a pathological condition given by a dysfunction located in the individual. In this picture, a person exhibiting PDB is psychologically flawed. It is an individualist, mechanistic conception of mental disorder, entrenched in a medical model of mental health; see Kendell (1975, 2001) and Wakefield (1992a,b) for two different versions of such a conception of mental disorder, which underlies some widely accepted diagnostic practices and manuals – including the DSM-5 (American Psychiatric Association, 2013) and some emergent trends in clinical and developmental psychology (e.g., Frick, 2016).

^2^In his experiments, Garfinkel (1984) demonstrated that people systematically treat one another’s actions as produced by morally accountable, choosing agents. As we unpack later, our moral (without quotation marks) view on PDB is also informed by Taylor’s notion of *strong evaluation* as the mark of the moral (Taylor, 1989, 2004; Chernilo, 2017). While we would not see all disruptive behavior (e.g., snoring) as morally informed, accepting such would only strengthen our point.

^3^For brevity’s sake, we focus here on normalization, as it is the most (although certainly not the only) pertinent to our argument. The other pivotal method is *surveillance* (Foucault, 1995).

^4^Parsons borrowed from Durkheim a similar way of reasoning to account for how institutions can be maintained non-coercively. Parsons (1968) argued that institutionalization understood as individual internalization of norms (e.g., role-bounded expectations, cultural values) was key to integrate otherwise multiple egoistic/instrumental interests.

^5^The postdisciplinarian idea of different but equally legitimate forms of life must not be conflated with radical, naive epistemic or moral relativism (roughly, the idea that there are no rational standards to establish facts and principles and, to that extent, each has “their own truth”). The challenge of coordinating multiple moral orders is, as McNamee (2018, p. 361) recently put it, “far from anything goes.” To the extent that the problems underlying the establishment of facts and principles are complex, both philosophically/theoretically (see, e.g., Blackburn, 2006) and practically in everyday life (see, e.g., Garfinkel, 1984), they should not be brushed aside by adopting uncritically the simplistic views behind radical, naive relativism. So far as we are concerned, much more promising approaches to overcome the dominance of unique, “disciplinary” moral cannons can be found (to name a few) in Fraser’s cultural justice (Fraser and Butler, 2016), Taylor’s (1997) politics of recognition, Gadamer (2004) fusion of horizons, Fricker’s (2007) epistemic justice, and Freire’s (2005) invitation to humbly, horizontally learn from each other.

^6^This is not to say that psychiatrically diagnosing individuals is *de facto* decreasing (it is not!), but that its legitimacy (as an acceptable, non-objectionable social practice) is declining.

^7^In 2006 elementary and high school students in Chile organized a strike (aka “March of the Penguins”) paralyzing the country’s educational system with great support from the general population. The same year, the “Great American Boycott” of schools and businesses by immigrants took place in the US. Both are two other good examples of major disruption made by people who suffer from some degree of marginalization or exclusion.

^8^Note that cultural anomy is not a direct result of postdisciplinary society, but of code juxtaposition. Nor is it the case that all people feel confused with juxtaposition. Actually, those who readily embrace it end up endorsing the sort of radical relativism we objected to in a previous note. So neither cultural anomy nor moral or epistemic relativism are necessary features of a postdisciplinary society, although both have tended to proliferate. In the last part of this paper we suggest a certain way to deal with postdisciplinary moral conflict (and PDB) that, unlike mindless code juxtaposition, keeps us away from the disorientation of cultural anomy as well as from the serious philosophical problems of radical relativism.

^9^We use the inverted commas because giving someone a drug is not tantamount to medicating that person unless the person does have a medical condition which can be so treated. But parents, especially among those who are reluctant to give the drug to their children, may certainly doubt that the so-called “attention deficit disorders” are in fact medical conditions – and, to that extent, that the drugs count as “medication” (more on this in Gaete, 2008; Ferretti and Gaete, 2020).

^10^Taylor has extensively elaborated on this type of normative meta-assessments, which he calls strong evaluations, and which he considers the mark of the moral (e.g., Taylor, 1989, 2004; see also Chernilo, 2017).

^11^This section owes a lot to Tomm’s ideas on reconciliation and forgiveness in family therapy. As far as we know, the distinction between hierarchical/juxtaposing approaches to conflict has not been documented but is part of what I (JG) have learned in the oral tradition of family therapy. I learned it from Tomm, who indicated might have learned this from Gianfranco Cecchin.

^12^Some may think assuming one’s own view immediately as less legitimate than the other’s might be a fourth option here. However, we would think that the person will not feel the behavior “offensive,” or at least would not engage in enduring conflict if in their immediate experience is informed by the judgment “I am (probably) wrong.” Hence, this fourth scenario is incompatible with the experience of PDB.

^13^Aristegui (1999, p. 309) addressed the issue of understanding clients’ meanings in psychotherapy invoking the hermeneutic principle of charity (elaborated by philosopher D. Davidson, who suggested that to understand a foreing language, “we need to assume that native speakers speak correctly”; our translation).

^14^People may find pragmatic solutions to their moral conflicts without necessarily agreeing that both parties hold legitimate views (i.e., without achieving juxtaposition). Unlike people reaching compromising tolerance, they may simply give up determining whether their views should be juxtaposed (or hierarchized), taking perhaps a stance of indefinite – rather than transforming – tentativeness. We see the three scenarios we describe in this section as paradigmatic situations offering more promising alternatives to deal with PDB in a postdisciplinary context, and not as exhaustive possibilities ensuing from bracketing moral judgments.
